# Melatonin and Its Homologs Induce Immune Responses *via* Receptors trP47363-trP13076 in *Nicotiana benthamiana*

**DOI:** 10.3389/fpls.2021.691835

**Published:** 2021-06-30

**Authors:** Mengmeng Kong, Tao Sheng, Jing Liang, Qurban Ali, Qin Gu, Huijun Wu, Jian Chen, Jia Liu, Xuewen Gao

**Affiliations:** ^1^Key Laboratory of Monitoring and Management of Crop Diseases and Pest Insects, Department of Plant Pathology, College of Plant Protection, Ministry of Education, Nanjing Agricultural University, Nanjing, China; ^2^International Genome Center, Jiangsu University, Zhenjiang, China; ^3^Chongqing Key Laboratory of Economic Plant Biotechnology, College of Landscape Architecture and Life Science/Institute of Special Plants, Chongqing University of Arts and Sciences, Chongqing, China

**Keywords:** melatonin, 5-methoxytryptamin, 5-methoxyindole, disease resistance, *Nicotiana benthamiana*, stomatal closure, salicylic acid, transmembrane receptor

## Abstract

Melatonin (N-acetyl-5-methoxytryptamine), a naturally occurring small molecule, can protect plants against abiotic stress after exogenous treatmenting with it. It is not known if melatonin homologs, such as 5-methoxytryptamine and 5-methoxyindole, that are easy and more cost-effective to synthesize can stimulate the plant immune system in the same manner as melatonin. In the present study, we assessed the biological activity of the melatonin homologs, 5-methoxytryptamin and 5-methoxyindole. The results showed that melatonin and its homologs all induced disease resistance against *Phytophthora nicotianae* in *Nicotiana benthamiana* plants. The application of all three compounds also induced stomatal closure and the production of reactive oxygen species. Gene expression analysis indicated that the expression of genes involved in hydrogen peroxide (H_2_O_2_), nitric oxide (NO) production, and salicylic acid (SA) biosynthesis was significantly upregulated by all three compounds. Four homologs of the melatonin receptors were identified by blasting search with the phytomelatonin receptor in *Arabidopsis*. Molecular docking studies were also used to identify four putative melatonin receptors in *N. benthamiana*. Further experimentation revealed that silencing of the melatonin receptors trP47363 and trP13076 in *N. benthamiana* compromised the induction of stomatal closure, *PR-1a* gene expression and SA accumulation by all three compounds. Collectively, our data indicate that the induction of defense responses in *N. benthamiana* by melatonin, 5-methoxytryptamine, and 5-methoxyindole involves the melatonin receptors trP47363 and trP13076.

## Introduction

Plants have evolved two innate immunity systems that respond to a broad range of microorganisms (Zuppini et al., 2004; Zhang et al., 2010; Jian et al., 2021). In the early phase of the plant immunity response, pathogen-associated molecular patterns (PAMPs) and pathogen elicitors can directly induce a defense response in plants (Chen et al., 2015). Elicitors may be secreted by the microorganism attempting to infect a host plant or substances released by hydrolytic enzymes produced by the pathogen or plant. Elicitors include a variety of compounds secreted by phytopathogens, including proteins, glycol-proteins, synthetic molecules, glycans, and lipids (Zhang et al., 2009). Plant cells respond to the elicitors by an influx in calcium ions, as well as the production of active oxygen species (AOS) and nitric oxide (NO). These induced molecules regulate many processes and interconnect different pathways to amplify and generate a physiological response by the host plant, such as a hypersensitive response and stomatal closure, through the induction of transcriptional and metabolic changes (Garcia-Brugger et al., 2006; Zhang et al., 2010). The various downstream responses that are triggered are not independent but rather overlap, including the induced expression of pathogenesis-related (*PR*) genes, phytohormone homeostasis regulation, synthesis of reactive oxygen species (ROS), and secondary metabolite accumulation (La Camera et al., 2004; Pieterse et al., 2009; Feng and Shan, 2014).

Melatonin (N-acetyl-5methoxytryptamine), originally discovered in the 1950s in the pineal gland, represents biological molecules that have been extensively studied. Melatonin (MT) has been shown to possess a broad spectrum of biological functions in both animals and plants (Lerner et al., 1958; Arnao and Hernández-Ruiz, 2006; Reiter et al., 2010; Liang et al., 2015; Zhang et al., 2015). For example, MT is considered a indole that can be potentially used to combat viral diseases, such as severe acute respiratory syndrome and West Nile virus (Bonilla et al., 2004), and has been shown to regulate circadian rhythms, the immune system, and ROS signaling, as well as sleep, food intake, mood, and body temperature in humans (Dollins et al., 1994; Rodriguez et al., 2004; Brainard et al., 2011; Carrillo-Vico et al., 2013). MT can also induce host resistance in plants to pathogens, such as *Alternaria* spp., *Pseudomonas syringae* pv. *tomato* DC3000, and *Fusarium* spp. (Lee et al., 2014; Arnao and Hernández-Ruiz, 2015). MT was first identified in plants in 1995 (Dubbels et al., 1995; Hattori et al., 1995) and has since received increased attention due to its common presence and versatile functions in the plant kingdom. For example, MT has been reported to alleviate abiotic stresses (cold, heat, drought, salt, and heavy metals) in plants (Shi et al., 2015b; Wei et al., 2015; Li et al., 2016b; Ding et al., 2018). MT in plants has also been reported to function as a growth regulator (Murch and Saxena, 2002; Arnao and Hernández-Ruiz, 2006) regulate seed germination, root development, and provide photoprotection, as well as regulate flowering, leaf senescence, seed yield, and fruit ripening (Wang et al., 2012; Byeon and Back, 2014; Byeon et al., 2014a; Zhang et al., 2014; Sun et al., 2015). The role of MT in plant immunity has been investigated but only limited information is available on the activity of MT-related homologs and the identification of MT receptors in plants.

The biosynthetic pathway of MT in many different organisms, including amphibians, reptiles, and mammals is well established (Reiter, 1999; Falcón et al., 2009) and differs in several respects from MT biosynthesis in plants. In the MT biosynthesis pathway in vertebrates, tryptophan is converted into either 5-hydroxytryptophan by tryptophan 5-hydroxylase or tryptamine by a tryptophan decarboxylase. While the 5-hydroxytryptophan pathway is predominant in animals, the tryptamine pathway is dominant in rice (*Oryza sativa*; Park et al., 2012). The MT biosynthetic pathway is not regulated in the same manner in plants and animals. The biosynthetic pathway for MT in plants is more complex, relative to vertebrates; however, only limited data are available on MT biosynthesis in plants (Tan et al., 2013). All the enzymes in the MT biosynthetic pathway in rice (*O. sativa*) have been recently characterized and localized (Byeon et al., 2014b). In contrast to biosynthesis, the production of synthetic MT is a common practice and results in the synthesis of high purity MT, which serves as the primary source of MT for therapeutic use in humans. MT is commercially synthesized in a four-step reaction, using 5-methoxyindole as the main starting material (Cheng-Hong et al., 2009).

In the present study, we investigated the effect of 5-methoxytryptamine and 5-methoxyindole on plant defense response. Results indicated that 5-methoxytryptamine and 5-methoxyindole can induce a resistance response in *Nicotiana benthamiana* against *Phytophthora nicotianae* similar to melatonin. The synthetic compounds 5-methoxytryptamine and 5-methoxyindole also induced stomatal closure and ROS production and activated the SA signaling pathway, which has not been investigated in other species. We also identified four putative melatonin receptors, trP47363, trP13076, trP49122, and trP40966 in *N. benthamiana* through bioinformatics prediction and molecular docking analyses. Finally, we found that the homologs receptors trP47363 and trP13076 act downstream of MT, 5-methoxytryptamine, and 5-methoxyindole in *N. benthamiana* through VIGS experiments. Collectively, the data suggest that the transmembrane receptors trP47363 and trP13076 function as receptors of MT, 5-methoxytryptamine and 5-methoxyindole to mediate plant defense response in *N. benthamiana*.

## Materials and Methods

### Fungal and Plant Growth Conditions

The original culture of *P. nicotianae* was provided by professor Daolong Dou (Nanjing Agricultural University, Jiangsu, China). A 0.6-cm-diameter plug containing *P. nicotianae* mycelium was placed at the center of V8 plates (200 ml V8 juice and 3.0 g CaCO_3_ were boiled in 1 l of water and solidified with 1.5% agar), and the plates were incubated at 25°C for 4 days. *N. benthamiana* seeds were surface sterilized in 95% (v/v) ethanol for 5 min, followed by a 5% (w/v) solution of sodium hypochlorite for 5 min. This was followed by five washes with double distilled water (ddH_2_O). Seeds were allowed to germinate in Petri plates containing solid (Murashige and Skoog 1962) medium. Seedlings were then transferred to pots containing sterilized vermiculite at a density of one per pot. Seedlings were incubated in a controlled environment growth chamber at 25°C under a 16 h/8 h light/dark cycle.

### The Effect of MT on Disease Severity in *Nicotiana benthamiana* Caused by *Phytophthora nicotianae*

Chitosan, MT, and its related compounds were dissolved in 99.7% (v/v) ethanol, then, diluted all the compounds to 50 μM using the sterilized water. The effect of 50 μM solutions of either MT, 5-methoxytryptamine, 5-methoxyindole, N-acetyltryptamine, tryptamine, indole, or chitosan of disease severity in *N. benthamiana* caused by *P. nicotiana* was assessed. Six-week-old *N. benthamiana* plants were treated with 50 μM solutions of MT, MT-homologs, and chitosan by watering the soil of seedlings with the prepared solutions. Control seedlings were treated with 0.005% (v/v) ethanol/water. Four hours after the treatments were applied, nine leaves from three plants were detached and inoculated with a 7 mm × 7 mm hyphal plug of *P. nicotianae* placed on the surface of the right side of each leaf, after inoculated samples were placed at 25°C. For disease symptoms were recorded after 48 h of incubation. Leaves were fixed in 95% ethanol, and the disease severity was assessed by measuring the diameter of the *P. nicotianae* lesion. Each assay was repeated three times.

### Effect of Treatments on Stomatal Aperture

Stomatal apertures were measured as described by Chen et al. (2004). *N. benthamiana* plants were watered with MT, or its homologs after seedlings had fully opened their stomata in the light for 2–3 h. This was done to minimize the effect of extraneous factors on stomatal response. Plants were then watered with either 50 μM solutions of either MT, 5-methoxytryptamine, 5-methoxyindole, N-acetyltryptamine, tryptamine, indole, or chitosan. Measurements of stomatal apertures were recorded 3 h after the treatments were applied. Images of different stomatal aperture leaf strips were captured with an Olympus BX43 microscope and then measured using CellSens Standard Software (Tokyo, Japan). The diameter of 50 randomly selected stomata, which got from nine leaves and three plants, was measured. Each assay was repeated three times.

### 3,3'-Diaminobenzidine Staining for Hydrogen Peroxide

Leaves collected 6 h after treatment were incubated in PBS buffer (pH 7.4) containing 0.5% (w/v) diaminobenzidine (DAB) for 8 h at 25°C in the light as described in Samuel et al. (2005). Leaves were then boiled in 96% ethanol for 10 min to remove chlorophyll and any unreacted dye. The remaining brown precipitates results from the reduction of the DAB by hydrogen peroxide were then assessed. The intensity and pattern of H_2_O_2_ staining in the leaves were analyzed using Quantity One software (Bio-Rad, Milan, Italy).

### RNA Isolation and Reverse Transcription-Quantitative PCR

Total RNA was extracted using a Plant RNA Kit (Omega Bio-Tek, Norcross, GA, United States) according to the manufacturer’s instructions. First-strand cDNA was synthesized using Reverse Transcriptase (TaKaRa Bio Inc., Dalian, China) with oligo dT primers, and the resulting cDNA was used as a template for subsequent PCR amplification. RT-PCR products were resolved on an agarose gel to determine qualitative expression levels of target genes. RT-qPCR was performed using SYBR Premix Ex Taq (TaKaRa Bio Inc.) on a 7,500 Fast Real-Time PCR Detection System (Applied Biosystems, Foster, CA United States). Expression of the *N. benthamiana EF1a* gene was used to normalize gene expression in each sample.

### Virus-Induced Gene Silencing

The first phytomelatonin receptor (CAND2/PMTR1) was recently identified in *Arabidopsis thaliana* and was demonstrated to regulate the stomatal closure induced by MT (Jian et al., 2018). Four membrane proteins (trP47363, trP13076, trP49122, and trP40966) were identified in *N. benthamiana* by BLASTP analysis^1^ using *Arabidopsis* CAND2/PMTR1 as a query. Specific, short fragments (∼400 bp) of *NbtrP49122-trP40966*, *NbtrP47363-trP13076*, or *NbtrP47363-trP13076-trP49122-trP40966* were designed using the Sol Genomics VIGS tool.^2^ These fragments were PCR-amplified with primers introducing 5'end EcoRI and 3' end XhoI sites and were cloned into the pTRV2 vector between EcoRI and XhoI sites (Liu et al., 2002). Cotyledons of two-week-old *N. benthamiana* seedlings were infiltrated with a mixture of *Agrobacterium tumefaciens* GV3101 harboring pTRV1 (OD_600_ = 0.5) and pTRV2: Gene to be silenced (OD_600_ = 0.5) into cotyledons and grown for 4–5 weeks under short-day conditions (22°C, 11 h light/13 h dark). Gene silencing was confirmed by semi-quantitative amplification of the silenced target gene. Total RNA of *N. benthamiana* was extracted from a single leaf disk (8 mm in diameter) using a Plant RNA Kit (Omega Bio-Tek, Norcross, GA, United States). First-strand cDNA was synthesized using Reverse Transcriptase (TaKaRa Bio Inc., Dalian, China) with oligo dT primers, and the resulting cDNA was used as a template for subsequent PCR amplification.

### *Agrobacterium*-Mediated Transient Expression Analysis

*Agrobacterium tumefaciens* GV3101 was transformed with binary vector effector constructs by electroporation (2.2 kV/6 ms/Bio-Rad). Transformed cells were grown on L-agar plates with selective antibiotics for 2 days then inoculated and grown in liquid L-media with selective antibiotics. Overnight cultures were centrifuged at 5,000 rpm for 4 min, resuspended in agro-infiltration solution (10 mM MgCl_2_/10 mM MES) and adjusted to an OD_600_ = 0.4 before leaf infiltration with a blunt-end syringe.

### Measurement of Free SA Accumulation

Free SA was extracted from leaves and roots and quantified according to the method of Schuhegger et al. (2006).

### Statistical Analysis

Each experiment was conducted with a minimum of three independent replications. The data were analyzed by ANOVA, followed by Duncan’s multiple range test (*p* < 0.01), using SPSS software (SPSS Inc., Chicago, IL, United States).

## Results

### Effect of Melatonin and Its Homologs on Disease Severity in *Nicotiana benthamiana* Caused by *Phytophthora nicotianae*

**Figure 1:**
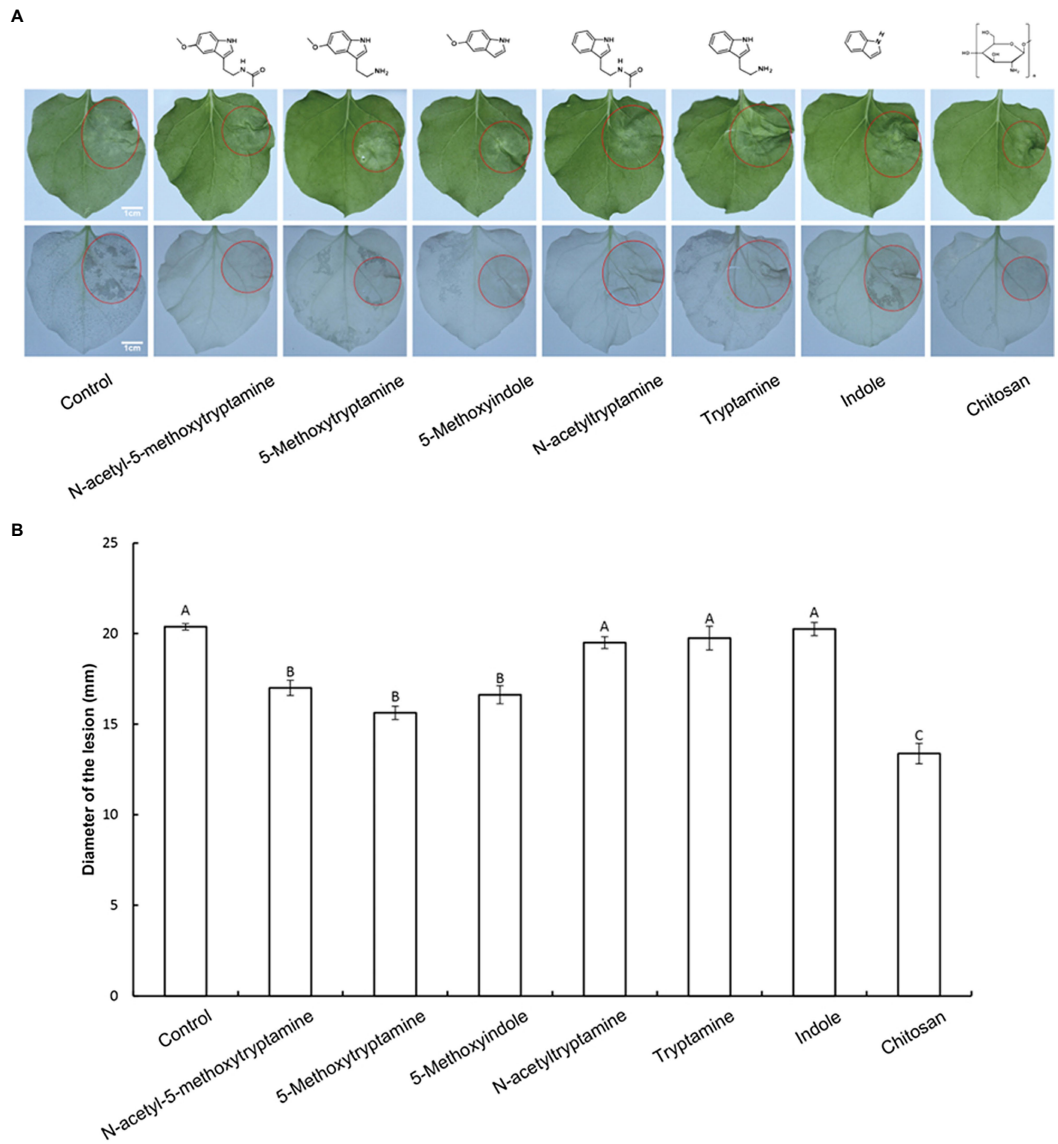
Effect of melatonin and its homologs on disease severity in *N. benthamiana* caused by *P. nicotianae*. **(A)** Disease symptoms in *N. benthamiana* leaves following pretreatment with 50 μM solutions of MT and MT-homologs and inoculate with *P. nicotianae*. Fully expanded *N. benthamiana* leaves were detached from plants 4 h after being watered with the test compounds. The leaves were placed in Petri dishes containing water-saturated filter paper and then inoculated with *P. nicotianae*. Photographs of the lesions were taken at 48 h post-inoculation. Water (control) was used as a negative control and chitosan was used as positive control. Ethanol-bleached *N. benthamiana* leaves were also photographed to more clearly illustrate the region of dead cells. **(B)** Average lesion diameter (red circles) in *N. benthamiana* leaves treated with different test compounds. Data represent the mean ± standard deviations (SDs) of three replicates (experiments). Columns with different letters indicate significant differences in lesion size as determined by a Duncan’s multiple range test.

MT plays an important role in plant protection against biotic stresses (Regodon et al., 2005; Carrillo-Vico et al., 2013; Vielma et al., 2014; Nabavi et al., 2019); however, it is expensive to produce, limiting its potential use in agriculture. Therefore, the impact of several MT-homologs on disease resistance in *N. benthamiana* against *P. nicotianae* was investigated. Water was used as a negative control and chitosan was used as a positive control. The experiment was designed to determine if 50 μM solutions of the treatment compounds could induce defense responses against *P. nicotianae* in *N. benthamiana*. Results indicated that the pretreatment of *N. benthamiana* plants with MT, 5-methoxytryptamine, and 5-methoxyindole significantly inhibited (*p* < 0.01) lesion size in inoculated leaves of *N. benthamiana* by 16.56–23.31%. Chitosan, used as a positive control, reduced lesion size by 34.36%, relative to the untreated control group. The other MT-homologs, N-acetyltryptamine, tryptamine, and indole, did not affect lesion size, relative to the untreated control (Figures 1A,B). Since MT, 5-methoxytryptamine, and 5-methoxyindole are based on a 5-methoxyindole backbone, while N-acetyltryptamine, tryptamine, and indole are based on indole, we postulated that 5-methoxyindole represents the functional backbone of MT.

### Melatonin, 5-methoxytryptamin, and 5-methoxyindole Induce Stomatal Closure in *Nicotiana benthamiana* Leaves

**Figure 2:**
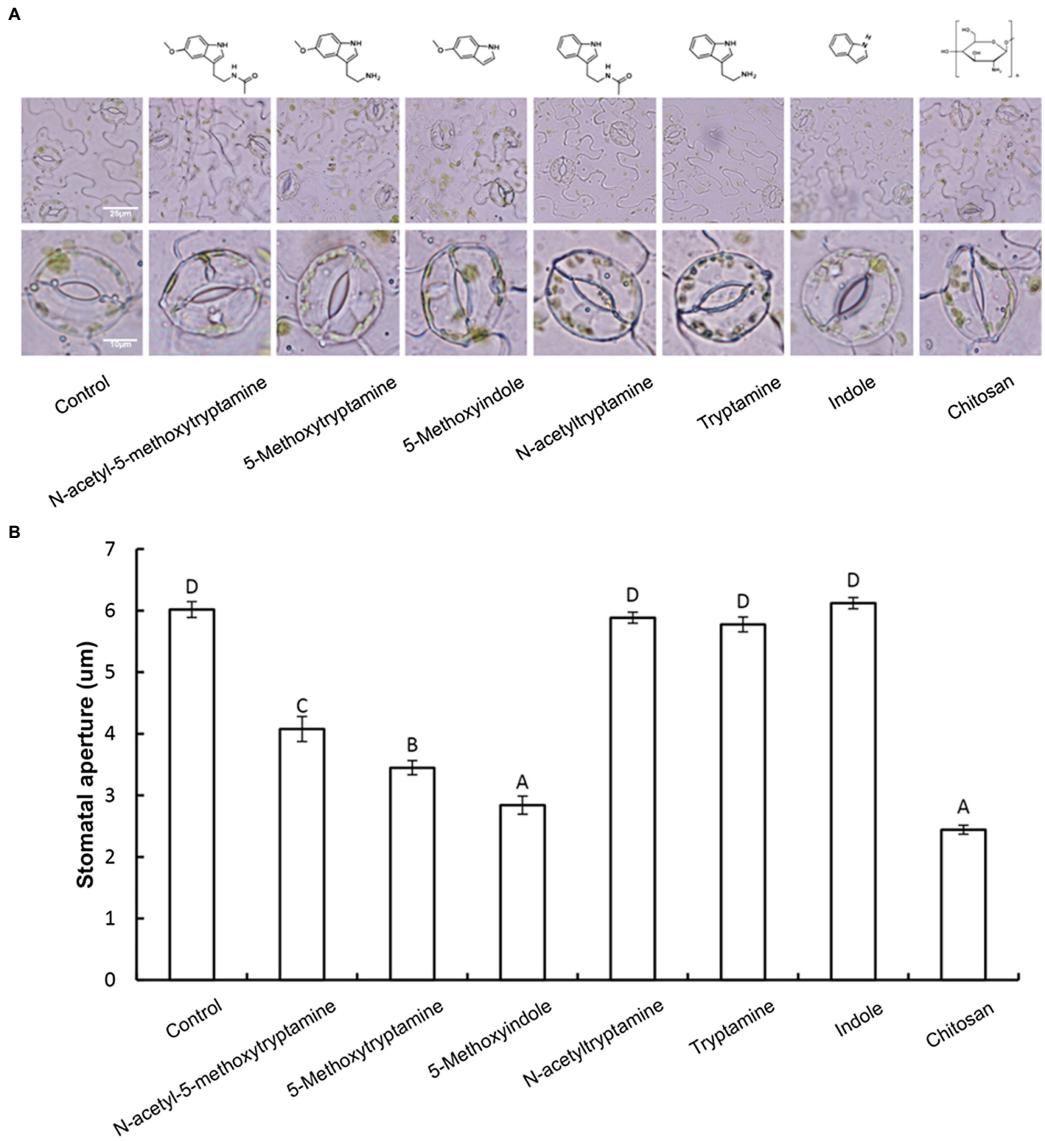
Melatonin, 5-methoxytryptamin, and 5-methoxyindole induce stomatal closure in *N. benthamiana* leaves. Measurements of stomatal apertures in *N. benthamiana* leaves 3 h after plants were treated with a 50 μM solution of MT, MT-homologs, and chitosan. Buffer-treated plants served as a control. 5-methoxytryptamin, 5-methoxyindole, and chitosan induced stomatal closure in *N. benthamiana* leaves, while the other test compounds and the buffer had no effect. **(A)** Representative micrographs of stomata in epidermal peels of treated plants 3 h after treatment with the test compounds. Lower panel of micrographs represents a higher magnification of individual stomata. **(B)** Stomatal data are the mean ± standard deviations (SDs; *n* = 150) of three independent experiments. Columns with different letters indicate significant differences in the aperture size of stomata of plants treated with the different test compounds as determined by a Duncan’s multiple range test.

Guard cells exhibit a classic innate immune response to both PAMP compounds and pathogens (Zhang et al., 2009). Elicitors, such as chitosan, have been reported to induce stomatal closure (Lee et al., 1999). Therefore, we hypothesized that MT and MT-homologs would also induce stomatal closure. To test this hypothesis, the aperture of guard cells in epidermal peels of *N. benthamiana* was examined and measured under a microscope after being treated with a buffer, or 50 μM solutions of MT or MT-homologs compared with positive control treated with Chitosan. Results indicated that 3 h after the treatment MT, 5-methoxytryptamin, and 5-methoxyindole induced stomatal closure, while N-acetyltryptamine, tryptamine, and indole had no effect (Figures 2A,B).

### Melatonin and Its Homologs Induce the H_2_O_2_ Accumulation in *Nicotiana benthamiana* Leaves

**Figure 3:**
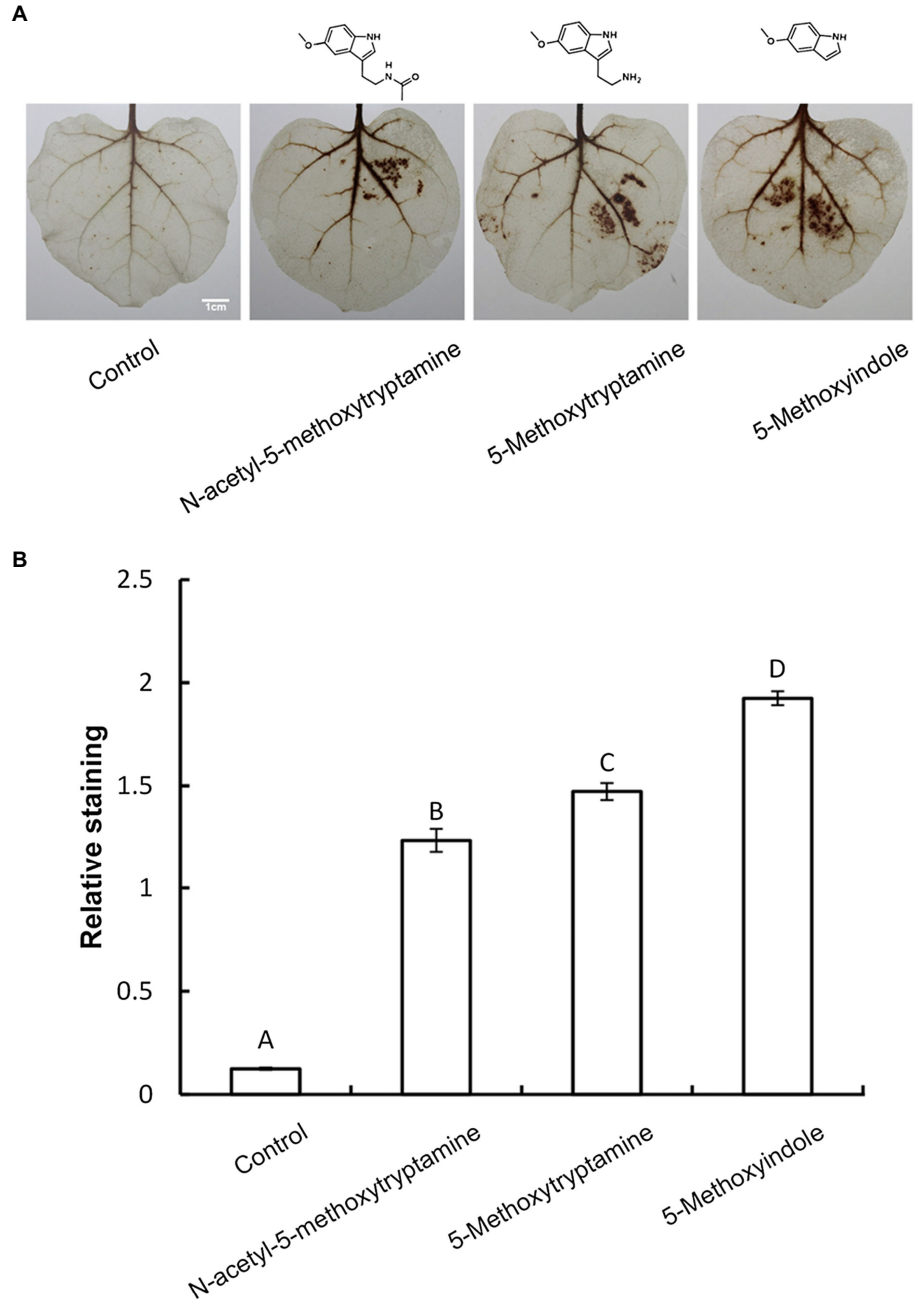
Melatonin and MT-homologs induce the H_2_O_2_ accumulation in *N. benthamiana* leaves. Detection of hydrogen peroxide (H_2_O_2_) in *N. benthamiana* leaves treated with various test compounds as determined by diaminobenzidine (DAB) staining. **(A)** Photographs of representative ethanol-bleached leaves 8 h after treatment with 50 μM solutions of MT, 5-methoxytryptamine, and 5-methoxyindole. **(B)** Quantitative scoring of staining in treated leaves. Data are the mean ± standard deviations (SDs; *n* = 27). Different letters indicate that the means are statistically significantly different at *p* < 0.01.

Several studies have demonstrated that H_2_O_2_ is involved in elicitor-inhibited stomatal opening and elicitor-enhanced stomatal closure (Lee et al., 1999). Therefore, H_2_O_2_ accumulation was measured in *N. benthamiana* leaves treated with MT, 5-methoxytryptamine, or 5-methoxyindole to determine if H_2_O_2_ was involved in the effect of these chemical compounds on stomatal closure. H_2_O_2_ accumulation was measured by DAB staining. Figure 3A is representative photographs showing the presence of DAB–H_2_O_2_ reaction product in *N. benthamiana* leaves at 8 h after treatment. Heavy brown precipitates, indicating H_2_O_2_ accumulation, were observed in leaves of plants treated with MT and MT-homologs, relative to the untreated control (Figure 3A). These observations were consistent with the measurement of relative DAB staining presented in Figure 3B.

### Effect of Melatonin, 5-methoxytryptamin, and 5-methoxyindoleregulate on the Expression of Genes Involved in H_2_O_2_ and NO Production

**Figure 4:**
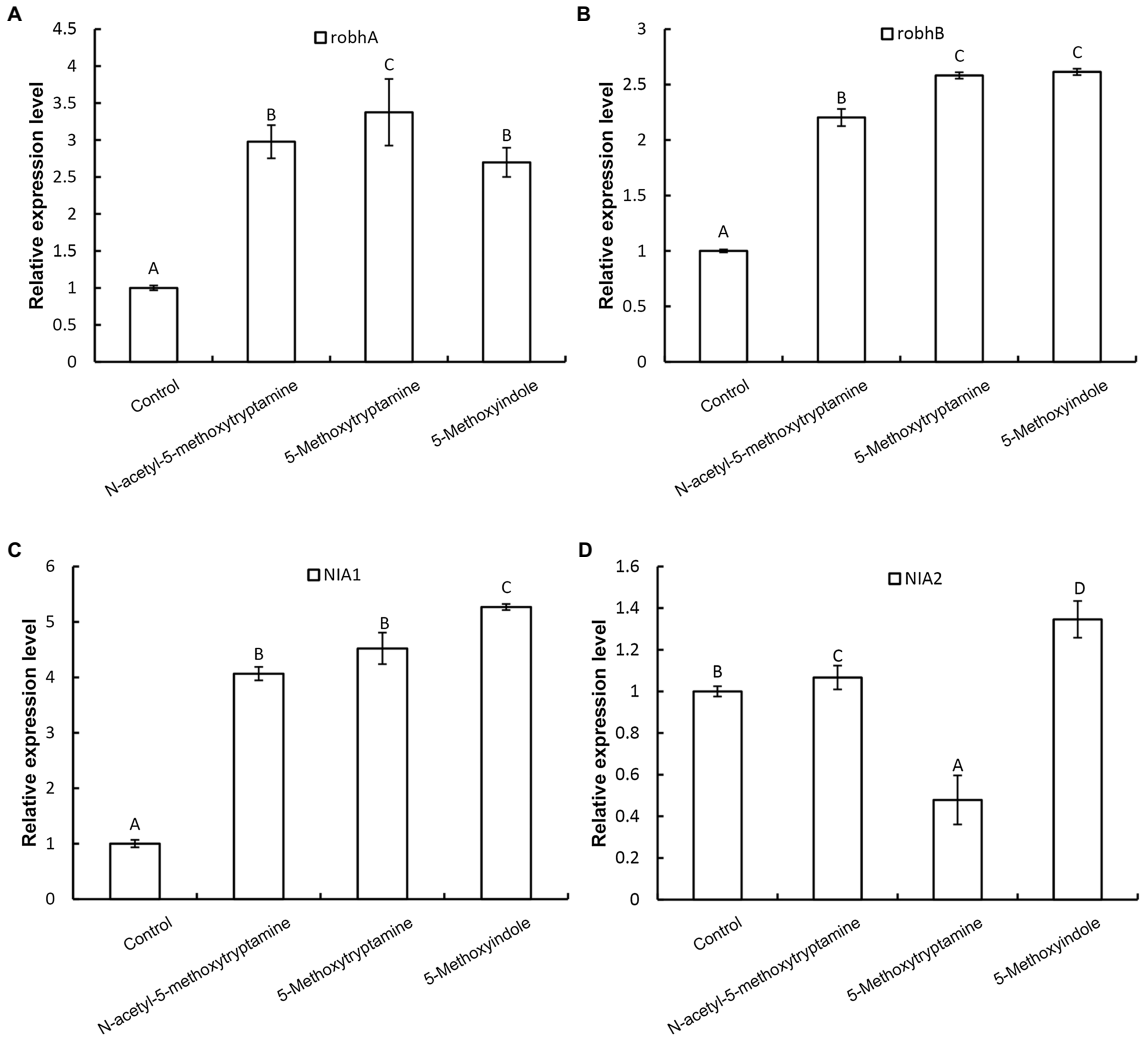
Effect of melatonin, 5-methoxytryptamin, and 5-methoxyindoleregulate on the expression of genes involved in H_2_O_2_ and NO production. The effect of MT and MT-homologs on the relative expression of genes involved in hydrogen peroxide and nitric oxide production. *N. benthamiana* plants were treated with water (control) or 50 μM solutions of MT, 5-methoxytryptamine, or 5-methoxyindole. The expression level of *robhA* **(A)**, *robhB* **(B)**, *NIA1* **(C)**, and *NIA2* **(D)** gene in leaves was determined by RT-qPCR. Data are mean ± standard deviation (SDs; *n* = 9) of three independent experiments. Columns with different letters indicate a significant difference (*p* ≤ 0.01) between the treatment groups as determined by a Duncan’s multiple range test.

Nitric oxide regulates innate immunity by functioning as a signaling molecule in a wide range of organisms including plants, especially in the regulation of stomatal guard cells (Ali et al., 2007; Asai and Yoshioka, 2009; Zhang et al., 2010). Rapid production of AOS, including H_2_O_2_, superoxide, and the highly reactive hydroxyl radical, is a characteristic component of the resistance response in plants, where AOS is believed to function as second messengers in the activation of resistance reactions, such as defense-related genes (Lee et al., 1999; Pasqualini et al., 2007). We hypothesized that the induction of disease resistance by MT, 5-methoxytryptamine, and 5-methoxyindole in plants is correlated with the upregulation of genes involved in H_2_O_2_ and NO production. Our results indicated that two genes involved in H_2_O_2_ production, *robhA* and *robhB*, were both significantly upregulated by MT, 5-methoxytryptamine, and 5-methoxyindole (Figures 4A,B). The expression of nitrate reductase genes, *NIA1* and *NIA2*, was also affected by the three tested compounds (Figures 4C,D). *NIA1* was upregulated by all three compounds, while the expression of *NIA2* was differentially impacted by the three compounds, being slightly induced by MT, more upregulated by 5-methoxyindole, and downregulated by 5-methoxytryptamine.

### Melatonin and Its Homologs Activate an SA-Mediated Signaling Pathway

**Figure 5:**
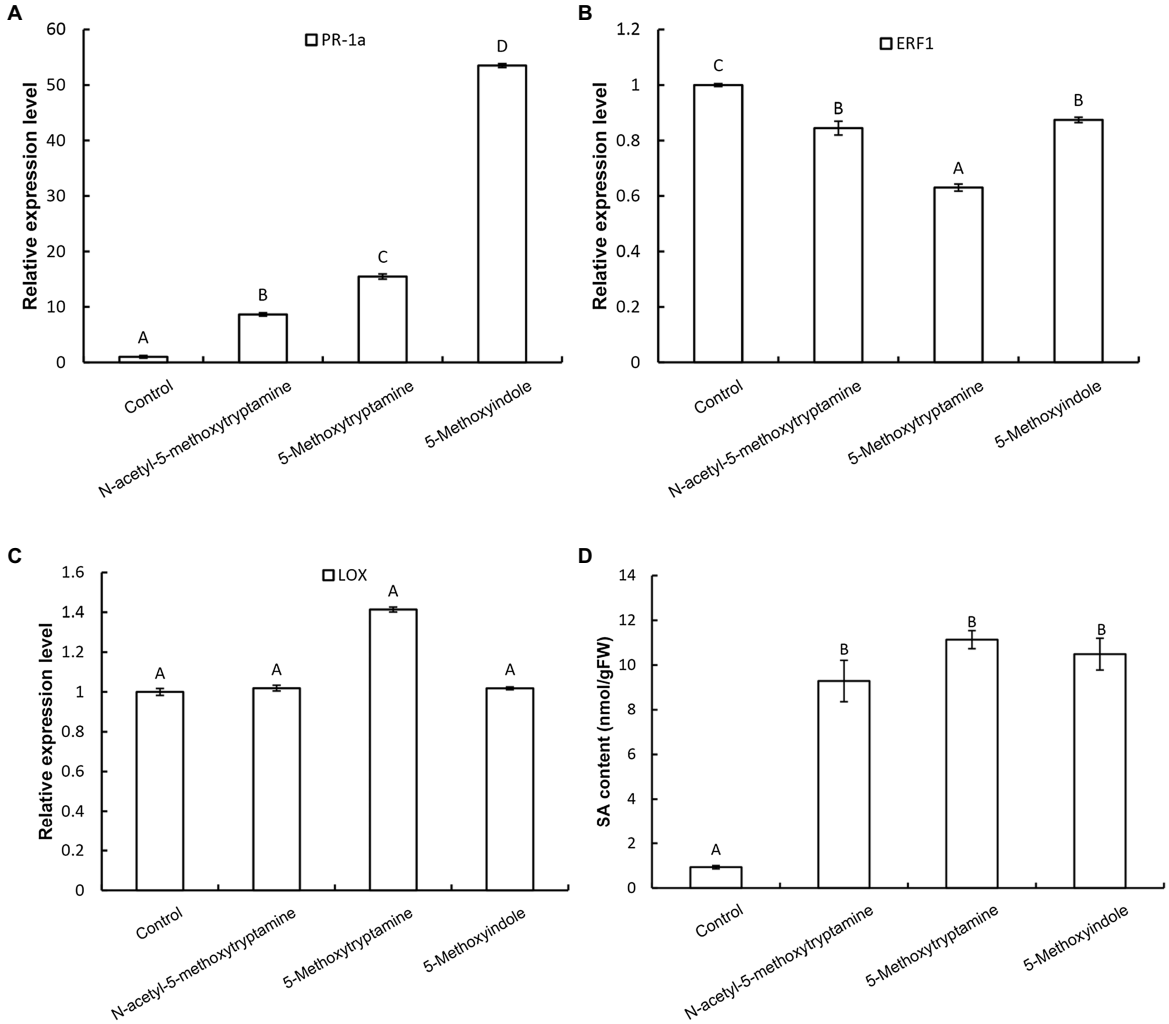
Melatonin and MT-homologs activate an SA-mediated signaling pathway. The effect of MT, 5-methoxytryptamine, and 5-methoxyindole on the expression of SA-, ethylene-, and JA-dependent marker genes in *N. benthamiana* leaves. *N. benthamiana* leaves were treated with 0.005% (v/v) ethanol/water (control) or 50 μM solutions of MT, 5-methoxytryptamine, or 5-methoxyindole. The relative expression of *PR-1a* **(A)**, *ERF1* **(B)**, and *LOX* **(C)** by RT-qPCR. Expression values were normalized to the levels of *EF-1a*. Data are the mean ± standard deviation (SDs; *n* = 9). Columns with different letters indicate a significant difference (*p* < 0.01) between the treatment groups as determined by a Duncan’s multiple range test. **(D)** Level of free SA in *N. benthamiana* leaves after treatment with MT, 5-methoxytryptamine, or 5-methoxyindole. Columns with different letters indicate a significant difference (*p* < 0.01) between the treatment groups as determined by a Duncan’s multiple range test. FW, fresh weight.

Plants utilize a broad range of defense mechanisms to respond and prevent invasion by pathogens. Systemic acquired resistance is associated with the accumulation of the hormone, salicylic acid (SA), while induced systemic resistance is mediated by jasmonic acid (JA) and ethylene (ET)-regulated pathways (Schuhegger et al., 2006; Zhu et al., 2014; Jian et al., 2019). To determine which signaling pathway is involved in the resistance of *N. benthamiana* to pathogens induced by MT and its homologs, the expression of the SA-responsive gene *PR-1a*, the JA synthesis-related gene *LOX*, and the ET-responsive gene *ERF1* in response to the application of these compounds. Results revealed that the expression of *PR-1a* is dramatically increased in *N. benthamiana* leaves 3 h after the application of MT, 5-methoxytryptamine, or 5-methoxyindole and that there was increased accumulation of free SA (Figures 5A,D), while *ERF1* was repressed (Figure 5B). Notably, the expression of *LOX* was not induced by any of the three tested compounds (Figure 5C).

### Bioinformatics Prediction of Four Membrane Receptors Potentially Involved in Binding Melatonin in *Nicotiana benthamiana*

**Figure 6:**
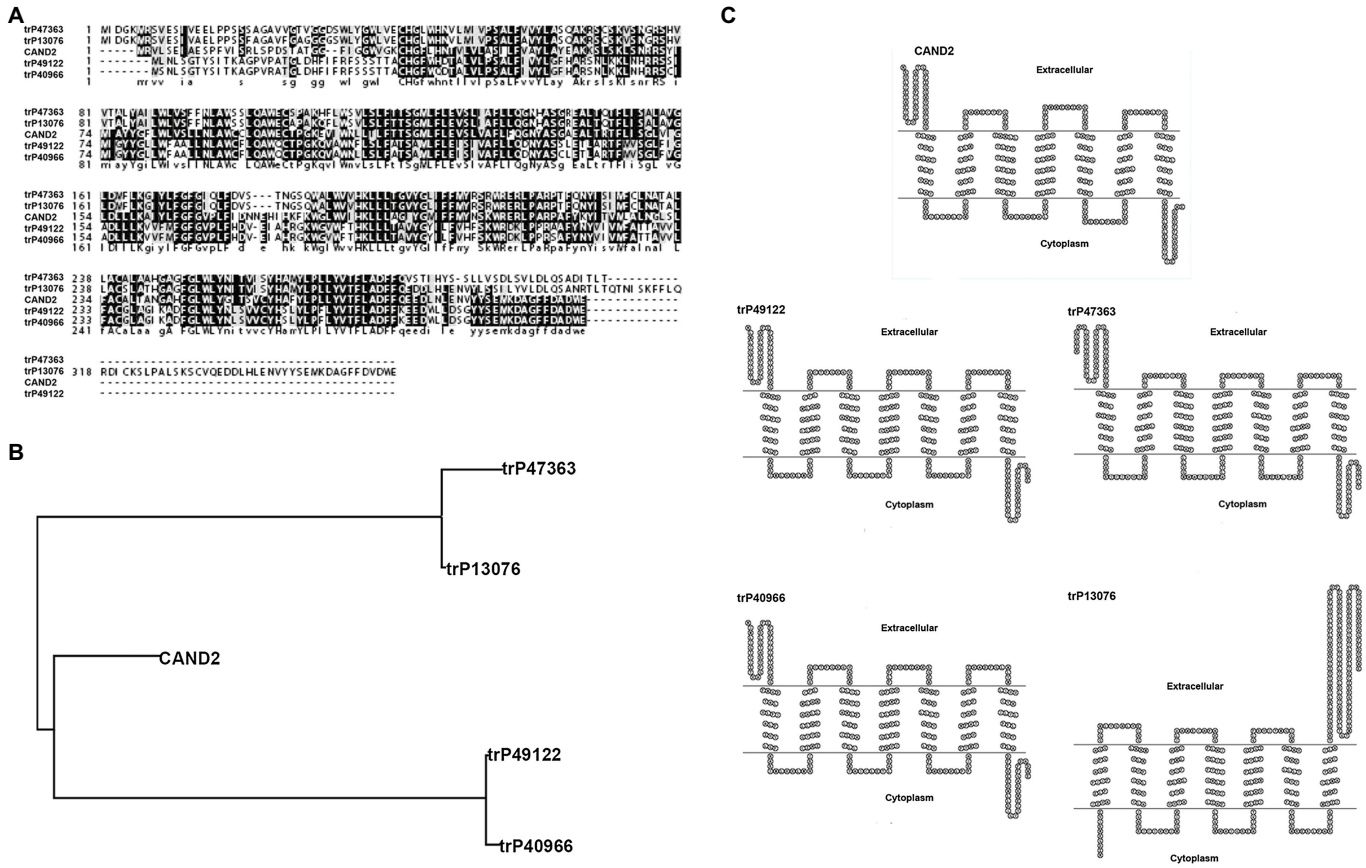
Bioinformatic prediction of four membrane receptors potentially involved in binding melatonin in *N. benthamiana*. Bioinformatic prediction and analysis of four putative transmembrane MT-receptor proteins in *N. benthamiana*. **(A)** Multiple sequence alignment of the known MT receptor in *Arabidopsis* (CAND2/PMTR1) with the putative MT-transmembrane receptors, trP47363, trP13076, trP49122, and trP40966 in *N. benthamiana*. Alignments were carried out using CLUSTAL (http://clustalw.genome.jp). Identical and similar amino acids are indicated by dark and light shading, respectively. **(B)** Evolutionary relationships between the known MT receptor (CAND2/PMTR1) in *Arabidopsis* and the putative MT receptors (trP47363, trP13076, trP49122, and trP40966) in *N. benthamiana*. The evolutionary history was inferred using the Neighbor-Joining method within the MEGA7 program. **(C)** The predicted transmembrane structure of CAND2 and trP47363, trP13076, trP49122, and trP40966 as determined using HMMTOP software (http://www.sacs.ucsf.edu/cgi-bin/hmmtop.py). The bold, underlined amino acid sequence indicates the putative transmembrane region.

MT was identified in plants as early as 1995; however, the function and signaling pathway of this putative phytohormone are largely undetermined due to the lack of identification of its receptor. The first phytomelatonin receptor (CAND2/PMTR1), however, was recently identified in *A. thaliana* and was demonstrated to regulate the stomatal closure induced by MT (Jian et al., 2018). In the current study, four membrane receptors were identified in *N. benthamiana* by BLASTP analysis (see footnote 1) using *Arabidopsis* CAND2/PMTR1 as a query. The amino acid sequence homology between the MT receptor in *Arabidopsis* (CAND2/PMTR1) and the four predicted receptors in *N. benthamiana* (trP47363, trP13076, trP49122, and trP40966) was high (Figure 6A). Sequence analysis indicated that the highest homology was between *Arabidopsis* CAND2/PMTR1 and *N. benthamiana* trP49122 and trP40966, with approximately 62% identity at the amino acid level. The sequence similarity between CAND2/PMTR1 and the *N. benthamiana* proteins trP47363, trP13076 was approximately 51%. A phylogenetic analysis of the MT receptor from *Arabidopsis* (CAND2/PMTR1) and the four transmembrane proteins revealed two distinct evolutionary branches (Figure 6B). Notably, however, the MT receptor (CAND2) in *Arabidopsis* has a shorter branch length distance from the membrane proteins trP49122 and trP40966 in *N. benthamiana* than from the proteins trP47363 and trP13076. A comparison of the predicted transmembrane structure of CAND2 in *Arabidopsis* with the putative MT receptors in *N. benthamiana* indicated that the structure of CAND2 was more similar to trP49122, trP40966, and trP47363 than to trP13076 (Figure 6C).

### *In silico* Molecular Docking Analysis of the *Nicotiana benthamiana* Membrane Receptors and Melatonin and 5-methoxyindole

**Figure 7:**
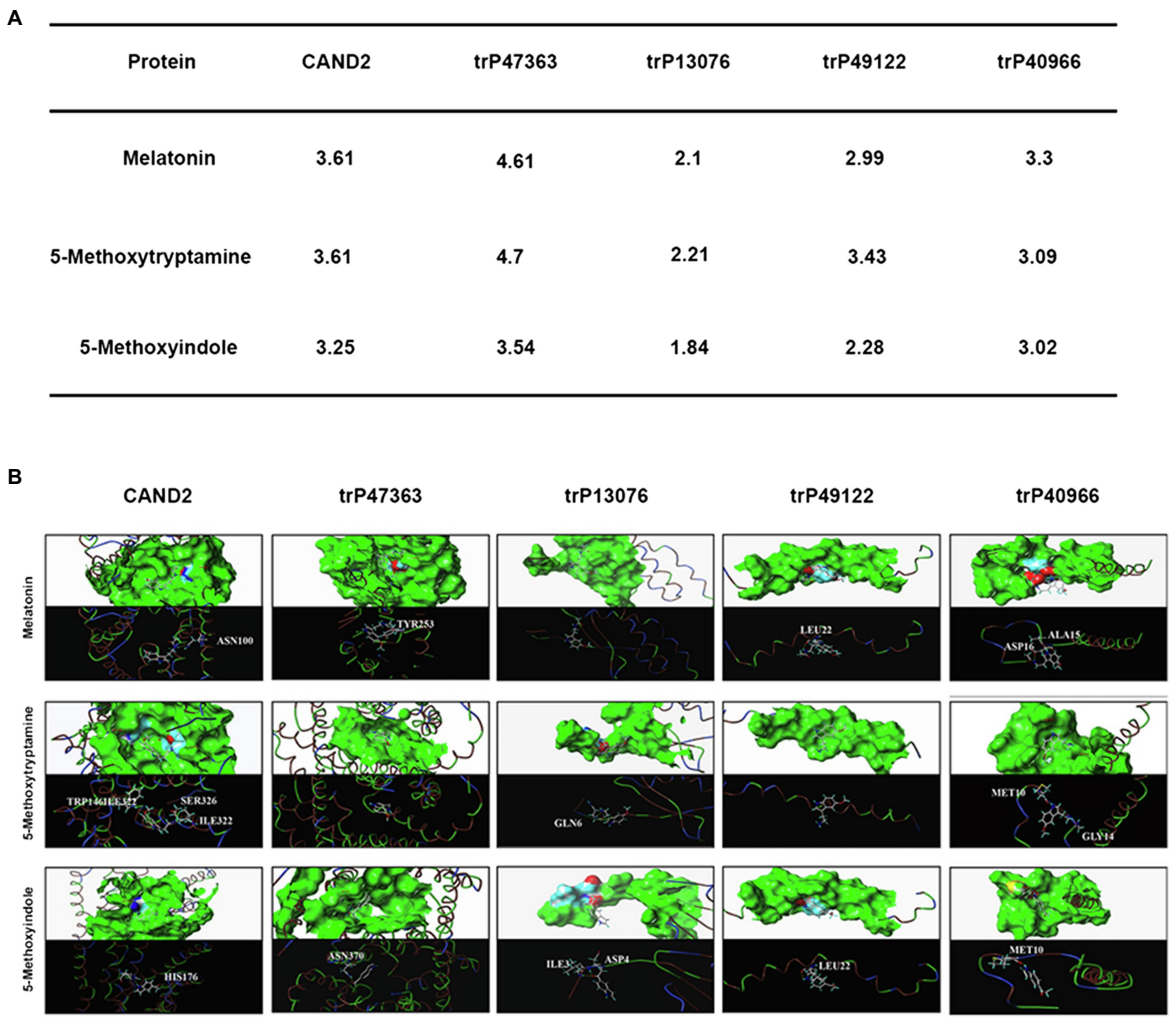
*In silico* molecular docking analysis of the *N. benthamiana* membrane receptors and melatonin and 5-methoxyindole. Interaction of *N. benthamiana* membrane receptors and CAND2 from *Arabidopsis* with MT and 5-methoxytryptamin and 5-methoxyindole **(A)**. Molecular docking analysis was conducted using SYBYL software to evaluate the binding model for the active binding pocket of the receptor proteins with MT, 5-methoxytryptamin, and 5-methoxyindole. The receptor proteins are displayed as colored ribbons, with the surface formed by the protein shown in green, and the amino acids capable of binding in blue, white, and red. MT, 5-methoxytryptamin, and 5-methoxyindole are represented as white, dark blue, and wine-red sticks. H-bonding interactions are indicated with dashed yellow lines **(B)**.

To further evaluate the molecular interaction between the putative transmembrane receptors identified in *N. benthamiana* and MT and 5-methoxytryptamine and 5-methoxyindole, a molecular docking study was conducted. Molecular docking analysis was conducted *in silico* using the surflex-dock program within SYBYL-X 2.0 software. The protein structure of the evaluated proteins was obtained from PDB.^3^ The scores obtained from the analysis indicate that MT can bind more efficiently to the receptor proteins than 5-methoxyindole, which lacks an amide bond. Since the structure of 5-methoxyindole is smaller than MT, the former molecule can enter the binding pocket formed by the receptor protein more flexibly and all of the crash scores for 5-methoxyindole are closer than of the crash scores of MT when comparisons are made for docking with the same receptor protein. The putative receptor proteins trP49122 and trP13076 do not exhibit an effective binding pocket, suggesting that even small molecules may not be able to interact with these proteins. CAND2 was confirmed to be a putative receptor protein for MT in *Arabidopsis* (Jian et al., 2018). The docking analysis resulted in a crash score of 3.61 for CAND2, which is considered to represent a good receptor protein for MT. As illustrated in Figure 7A, CAND2 can form an active bowl-like pocket and the oxygen atom at the amide group of ASN100 has one H-bonding interaction with the active hydrogen at the amide bond of MT (–NHCO– ------ H2NOC-ASN100).

Figure 7A also illustrates that trP47363 most likely represents the best receptor protein for MT in *N. benthamiana*, with a C score of 4.61 for docking with MT, which is the highest score indicated among the five receptor proteins. The binding of trP47363 with MT is illustrated in Figure 7B. Visual inspection indicates that MT is tightly embedded in the hole-shaped active binding pocket (Figure 7B), indicating that MT has a higher probability of interacting with trP47363 than with the other receptor proteins. The oxygen atom of the methoxy at the fifth position of the indole ring in MT provides one H-bonding interaction with the hydrogen atom of the hydroxyl group in TYR253 (-OCH ------HO-TYR253).

### Melatonin, 5-methoxytryptamin, and 5-methoxyindole Induce Stomatal Closure in *Nicotiana benthamiana* Leaves Involve the Transmembrane Receptors trP47363 and trP13076

**Figure 8:**
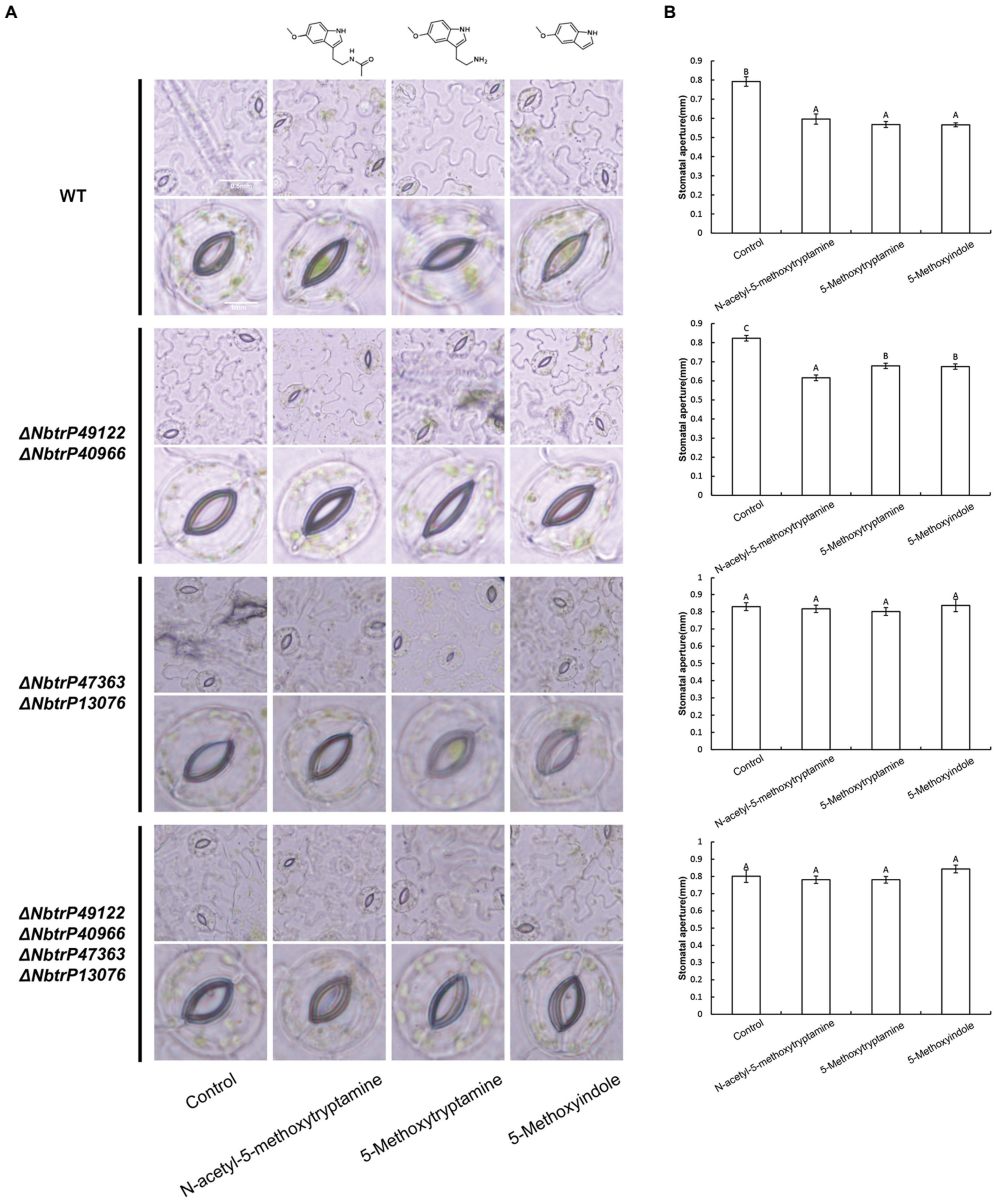
Melatonin, 5-methoxytryptamin, and 5-methoxyindole induce stomatal closure in *N. benthamiana* leaves involve the transmembrane receptors trP47363 and trP13076. MT-, 5-methoxytryptamin-, and 5-methoxyindole-induced stomatal closure in leaves of VIGS mutants of *N. benthamiana* revealing the role of the transmembrane receptors, trP47363 and trP13076. Two-week-old *N. benthamiana* plants were infiltrated with a mixture of *A. tumefaciens* Gv3101 strains carrying pTRV1 or pTRV2 constructs as described in the Materials and Methods. Five weeks later, plants were watered with 0.005% (v/v) ethanol/water (control) or 50 μM solutions of MT, 5-methoxytryptamine, or 5-methoxyindole. Stomata were observed under a microscope 3 h after the application of the treatment solutions. **(A)** Representative low- and high-magnification images. **(B)** Measurements of stomatal aperture. Data are the mean ± standard deviation (SD; *n* = 150). Columns with different letters indicate significant differences between the different treatment groups in the different VIGS lines as determined by a Duncan’s multiple range test.

The bioinformatics and molecular docking analyses provide fundamental information on MT and MT-homologs induced resistance in *N. benthamiana* to *P. nicotianae*. Further molecular and genetic evidence is needed, however, to support the role of the transmembrane receptors in MT and its homologs induced disease resistance. In this regard, knocking down the expression of key genes involved in plant immunity through virus-induced gene silencing (VIGS) has been widely used to evaluate the involvement of various defense components in plant immunity (Liu et al., 2002; Peart et al., 2002; Oh et al., 2014). We utilized VIGS to determine the need for the identified transmembrane receptors in MT, 5-methoxytryptamin, and 5-methoxyindole induced disease resistance in *N. benthamiana*. *N. benthamiana* plants were generated that were silenced for Δ*NbtrP49122*-Δ*NbtrP40966* (pTRV2:*NbtrP49122-trP40966*), Δ*NbtrP47363*-Δ*NbtrP13076* (pTRV2:*NbtrP47363-trP13076*), and Δ*NbtrP47363-trP13076-trP49122-trP40966*(pTRV2:*NbtrP47363-trP13076-trP49122-trP40966*) and were utilized together with control plants (pTRV2:EV). To first validate our experimental conditions, stomatal closure induced by MT, 5-methoxytryptamin, and 5-methoxyindole was evaluated in control and the VIGS mutants of *N. benthamiana*. Results indicated that MT, 5-methoxytryptamin, and 5-methoxyindole induced stomatal closure in EV- and *ΔNbtrP49122-trP40966*-plants but not in Δ*NbtrP47363-trP13076*- or in Δ*NbtrP47363-trP13076-trP49122-trP40966*-silenced *N. benthamiana* plants (Figures 8A,B). Collectively, the results indicate that the transmembrane receptors trP47363 and trP13076 are key components in the regulation of stomatal closure in *N. benthamiana* leaves by MT, 5-methoxytryptamin, and 5-methoxyindole.

### The Transmembrane Receptors trP47363 and trP13076 Play a Key Role in Melatonin, 5-methoxytryptamin, and 5-methoxyindole Activation of the SA Signaling Pathway

**Figure 9:**
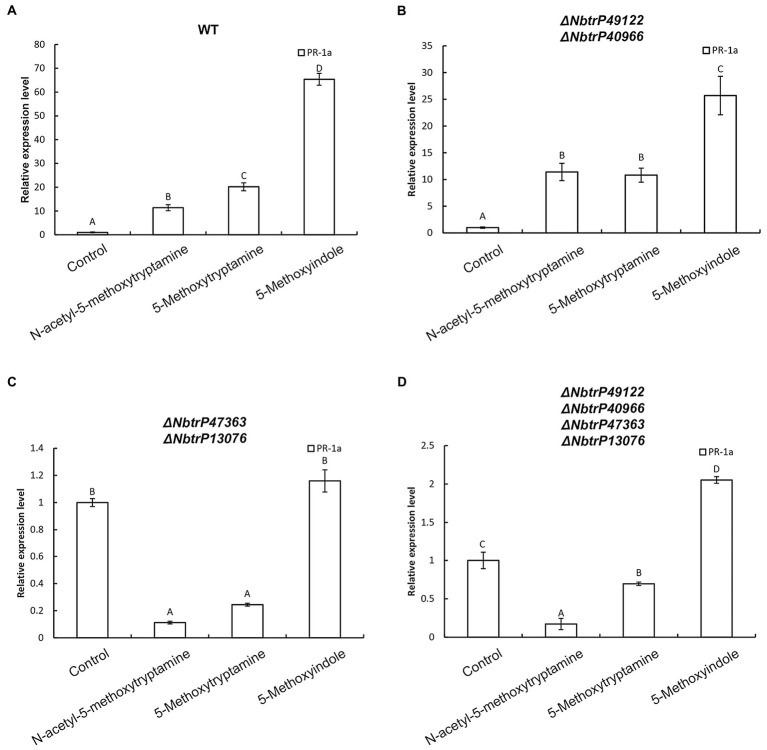
The transmembrane receptors trP47363 and trP13076 play a key role in melatonin, 5-methoxytryptamin, and 5-methoxyindole activation of the SA-signaling pathway. MT, 5-methoxytryptamine, and 5-methoxyindole induced expression of the SA marker gene, *PR-1a*, in leaves of wild-type and VIGS mutants of *N. benthamiana*. **(A)** RT-qPCR analysis of *PR-1a* gene expression in response to MT, 5-methoxytryptamine, and 5-methoxyindole treatments in wild-type plants. Values were normalized to levels of *EF-1a* expression. Values represent the mean relative expression level (fold-change) with expression levels in the control set to 1.0 ± standard deviation (SD; *n* = 9). **(B)** RT-qPCR analysis of MT, 5-methoxytryptamin, and 5-methoxyindole induced *PR-1a* gene expression in the mutant, Δ*NbtrP49122-trP40966*. (**C**,**D**) RT-qPCR analysis of MT, 5-methoxytryptamine, and 5-methoxyindole induced expression of *PR-1a* in leaves of the mutant lines, *ΔNbtrP47363-trP13076* and *ΔNbtrP47363-trP13076-trP49122-trP40966*.

Our results indicated that MT, 5-methoxytryptamin, and 5-methoxyindole can significantly upregulate *PR-1a* gene expression in wild *N. benthamiana* plants and the mutant line Δ*NbtrP49122-trP40966* (Figures 9A,B). Therefore, to determine which transmembrane receptor is involved in the expression of the SA marker gene, *PR-1a*, induced by MT, 5-methoxytryptamin, and 5-methoxyindole, the expression of *PR-1a* in different receptor mutants created by VIGS was assayed. Interestingly, MT- and 5-methoxytryptamine-induced *PR-1a* expression was significantly lower than in the control in Δ*NbtrP447363-trP13076* and *ΔNbtrP47363-trP13076-trP49122-trP40966* (Figures 9C,D). Additionally, 5-methoxyindole-induced expression of *PR-1a* was only slightly higher than MT-induced expression, or unchanged, in *ΔNbtrP447363-trP13076* and *ΔNbtrP47363-trP13076-trP49122-trP40966* (Figures 9C,D). Collectively, the data indicate that MT, 5-methoxytryptamine, and 5-methoxyindole activate the SA signaling pathway through the receptors trP47363 and trP13076.

### The Transmembrane Receptors trP47363 and trP13076 Are Involved in Melatonin-, 5-methoxytryptamin-, and 5-methoxyindole-Induced Accumulation of SA

**Figure 10:**
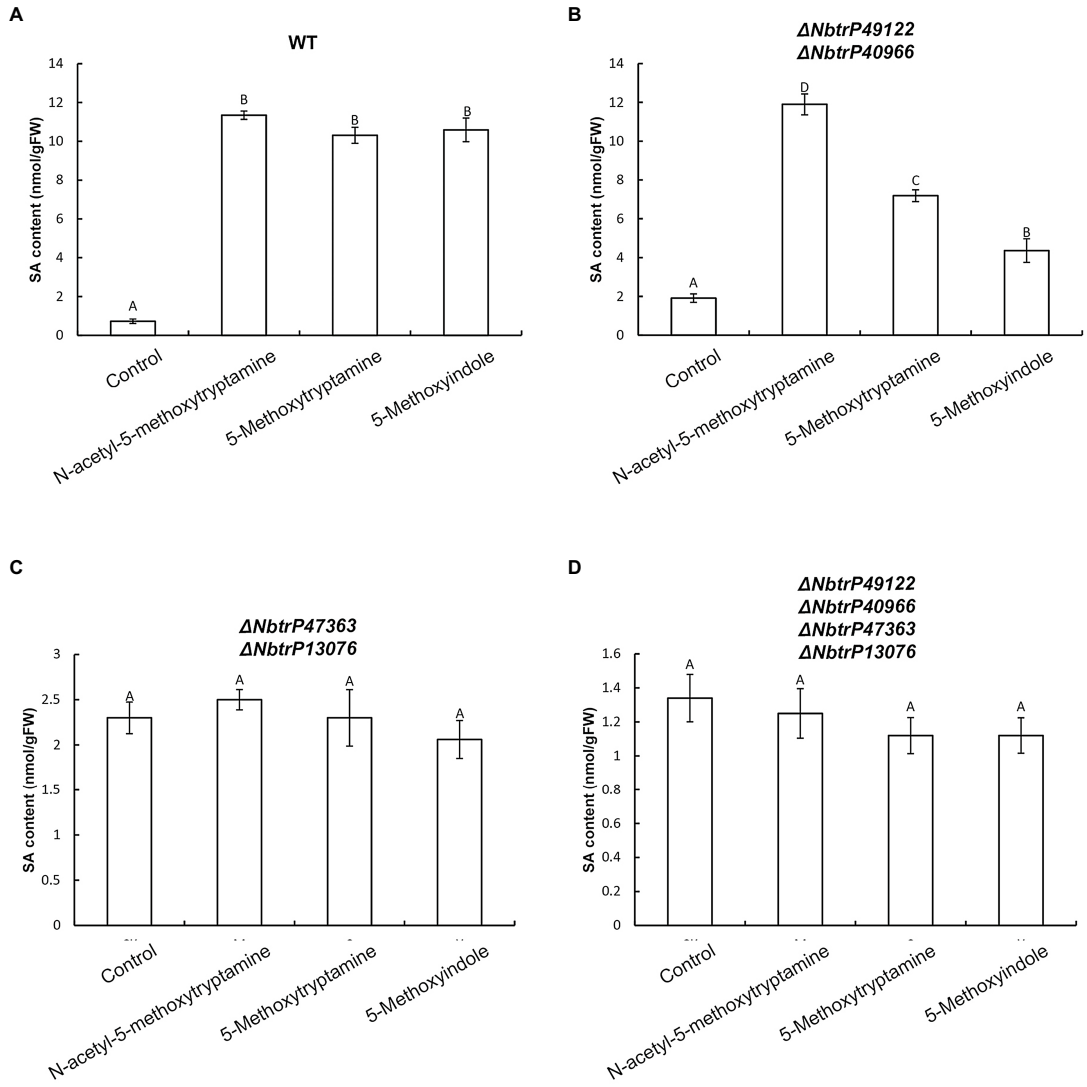
The transmembrane receptors trP47363and trP13076 are involved in melatonin-, 5-methoxytryptamin-, and 5-methoxyindole-induced accumulation of SA. SA levels in leaves of wild-type and mutant lines of *N. benthamiana* treated the MT, 5-methoxytryptomine, or 5-methoxyindole. Levels of free SA in *N. benthamiana* wild-type plants **(A)**, the Δ*NbtrP49122-trP40966* mutant **(B)**, the Δ*NbtrP47363-trP13076* mutant **(C)**, and the Δ*NbtrP47363-trP13076-trP49122-trP40966* mutant **(D)**. Data are the mean ± standard deviations (SDs; *n* = 3). Columns with different letters indicate significant differences between the treatment groups in the different lines of *N. benthamiana* as determined by a Duncan’s multiple range test (*p* < 0.01).

To determine if SA levels are altered in leaves of VIGS mutants of *N. benthamiana*, SA levels were measured in the different receptor mutants that were treated with MT, 5-methoxytryptamine, and 5-methoxyindole. Results indicated all three compounds significantly induced SA accumulation in wild-type plants and the Δ*NbtrP49122-trP40966* mutant (Figures 10A,B) but not in Δ*NbtrP447363-trP13076*orΔ*NbtrP47363-trP13076-trP49122-trP40966* mutants (Figures 10C,D). These results suggest that the transmembrane receptors trP47363 and trP13076 recognize MT, 5-methoxytryptamine, and 5-methoxyindole which are involved in the regulation of SA accumulation.

## Discussion

The role of melatonin (MT) in a variety of biological processes has been extensively investigated in animal systems and demonstrated to play a role in the regulation of circadian rhythms, the immune system, induction of ROS, sleep, food intake, mood, and body temperature (Dollins et al., 1994; Rodriguez et al., 2004; Brainard et al., 2011; Carrillo-Vico et al., 2013). In plants, MT has been reported to play a role in the regulation of seed germination, root development, photoprotection, flowering, leaf senescence, seed yield, and fruit ripening (Wang et al., 2012; Byeon and Back, 2014; Byeon et al., 2014b; Zhang et al., 2014; Sun et al., 2015). Studies have also implicated MT in plant immunity (Yin et al., 2013; Lee et al., 2015; Shi et al., 2015c,d; Mandal et al., 2018). While a variety of MT-homologs exist, which are more cost-effective to produce, it is not known if these MT-homologs, such as 5-methoxytryptamin and 5-methoxyindole can induce a defense response in plants. In the present study, we demonstrated that both 5-methoxytryptamin and 5-methoxyindole can induce the same defense response as MT in leaves of *N. benthamiana*. The results also indicate that the MT-homolog-induced defense response occurs through the activation of SA signaling. MT, 5-methoxytryptamine, and 5-methoxyindole induced disease resistance against *P. nicotianae* in leaves of *N. benthamiana* plants, and also induced stomatal closure through the activation of H_2_O_2_ production and SA accumulation. Based on these results, we suggest that both 5-methoxytryptamin and 5-methoxyindole can serve as cost-effective alternatives to the use of MT to induce a disease resistance response in plants.

NO and H_2_O_2_, as important components of signal transduction pathways, regulate a large number of physiological functions in biological systems. Stomatal closure occurs in response to physiological and stress stimuli (Bright et al., 2006; Garcia-Brugger et al., 2006), and numerous studies have shown that various elicitors can induce reactive oxygen bursts to limit the expansion of invasive pathogens (Zhang et al., 2009, 2010). MT has also been shown to induce increases in H_2_O_2_ production in *Arabidopsis* (Jian et al., 2018). In the present study, we found that H_2_O_2_, one of the most well-characterized plants signaling molecules, is involved in the MT, 5-methoxytryptamine, and 5-methoxyindole induction of systemic resistance in *N. benthamiana* against *Phytophthora parasitica* var. *nicotianae* (Figure 1). Our study also demonstrated that guard cells respond to MT, 5-methoxytryptamine, and 5-methoxyindole generate H_2_O_2_ and undergo a narrowing of their stomatal apertures (Figure 2). We also confirmed that MT, 5-methoxytryptamine, and 5-methoxyindole can stimulate the accumulation of both H_2_O_2_ and SA in *N. benthamiana* leaves (Figures 3, 5). These observations suggest that MT, 5-methoxytryptamine, and 5-methoxyindole could be considered as potential candidates for a new group of general elicitors of plant defense.

Disease resistance in plants is a highly regulated phenomenon that depends on several hormone-regulated signaling pathways, including salicylic acid (SA), jasmonic acid (JA), and ethylene (ET), each of which is activated by a different set of biotic and abiotic stimuli (Schuhegger et al., 2006). Pretreatment of plants with different elicitors can render a plant more resistant to subsequent pathogen attack, and elicitors have been demonstrated to induce various plant defense responses, such as the hypersensitive response, pathogenesis-related (PR) gene expression, and cell wall stabilization (Lee et al., 1999). Peanut (*Arachis hypogaea*) leaves treated with chitosan, a well-documented elicitor, exhibit an increase in endogenous SA levels, as well as an increase in intercellular chitinase and glucanase activity (Sathiyabama and Balasubramanian, 1998). In the present study, SA, a well-characterized signaling molecule in plants, was shown to be involved in the systemic resistance induced by MT, 5-methoxytryptamine, and 5-methoxyindole, as evidenced by the upregulation of *PR-1a* and SA accumulation, both of which are considered as markers of the SA-dependent defense pathway (SAR; Yoshioka et al., 2009; Figure 5).

MT functions as a neurohormone in vertebrates. Four families of MT receptors have been identified in animals (Reppert et al., 1994, 1995; Nosjean et al., 2000; Levoye et al., 2006). Phytomelatonin was first identified in plants in 1995 (Dubbels et al., 1995; Hattori et al., 1995; Tassel et al., 1995). Relative to its role in vertebrates, the designation of phytomelatonin as a hormone in plants remains controversial partially due to the lack of identification of an MT receptor or a receptor-mediated signaling pathway, as well as the function of such a pathway. The first phytomelatonin receptor (CAND2/PMTR1) was recently identified in *A. thaliana* and shown to mediate MT-induced stomatal closure (Jian et al., 2018). In our present study, CAND2/PMTR1 was used as a query in a BLASTP analysis to identify four putative transmembrane receptors (trP47363, trP13076, trP49122, and trP40966) in *N. benthamiana*. The resulting putative receptors identified in *N. benthamiana* exhibited a high amino acid sequence homology with the CAND2/PMTR1 of MT receptor in *A. thaliana* (Figure 6A). A phylogenetic tree and transmembrane structure prediction between the MT receptor in *A. thaliana* (CAND2/PMTR1) and the four transmembrane proteins in *N. benthamiana* were also similar (Figures 6B,C).

An in silico molecular docking analysis was also conducted to further evaluate the molecular interaction between the transmembrane receptors and MT, 5-methoxytryptamine, and 5-methoxyindole in *N. benthamiana*. As shown in Figure 7, trP47363 appears to be the best MT receptor protein in *N. benthamiana* based on its C score (4.61) for docking with melatonin, which was the highest among the five analyzed proteins and revealed that MT and MT-homologs could be tightly embedded into a hole-shaped active binding pocket. We speculate that trP47363 represents a putative transmembrane protein that is most likely to recognize MT, 5-methoxytryptamine, and 5-methoxyindole to trigger the induction of an induced immunity response in *N. benthamiana*. We then measured the expression of the plant defense gene, *PR-1a*, and SA accumulation and their association with *stomatal closure in leaves VIGS mutants of N. benthamiana treated with* MT, 5-methoxytryptamine, or 5-methoxyindole. The VIGS mutants were constructed to silence the expression of the four transmembrane receptors. The collective results indicated that the putative transmembrane receptors trP47363 and trP13076 appear to be involved in the induced immunity response in *N. benthamiana*. This includes stomatal closure, *PR-1a* expression and SA accumulation induced by MT, 5-methoxytryptamine, and 5-methoxyindole (Figures 8–10). Based on these results, it appears that the transmembrane receptor trP47363 plays an important role in recognizing MT, 5-methoxytryptamine, and 5-methoxyindole, which then induces a plant defense response by activating the SA-mediated signaling pathway.

In conclusion, we evaluated the ability of MT and MT-homologs to induce a resistance response in *N. benthamiana* and the underlying mechanisms associated with the resistance response. We demonstrated that 5-methoxyindole represents the functional backbone in MT and can induce the same response in *N. benthamiana* as MT. We also identified putative MT receptors in *N. benthamiana* and demonstrated that MT, 5-methoxytryptamine, and 5-methoxyindole are recognized by the same putative receptors. Our study provides direct evidence that 5-methoxytryptamine and 5-methoxyindole can induce resistance in *N. benthamiana*, in the same manner as MT. The three compounds trigger stomatal closure, induce the production of ROS, as well as SA accumulation. Notably, the membrane receptors trP47363 and trP13076 recognize MT, 5-methoxytryptamine, and 5-methoxyindole in *N. benthamiana*. We provide evidence that 5-methoxytryptamine and 5-Methozyindole can be substituted for MT as an inducer of plant resistance. Thus, 5-methoxytryptamine and 5-Methozyindole have excellent commercial potential for their use in agricultural production due to their ability to induce a resistance response, and the low cost of production relative to synthetic MT.

## Data Availability Statement

The raw data supporting the conclusions of this article will be made available by the authors, without undue reservation.

## Author Contributions

XG, JC, and JLiu conceived and designed the study and revised the manuscript. MK designed the study, analyzed the data, and wrote and revised the manuscript. TS and JLia performed the experiments, processed the data, and provided the suggestions. QA, QG, and HW revised the manuscript. All authors discussed the results and commented on the manuscript.

### Conflict of Interest

The authors declare that the research was conducted in the absence of any commercial or financial relationships that could be construed as a potential conflict of interest.

## Footnotes

^1^https://sefapps02.qut.edu.au/blast/blast_link2.cgi

^2^vigs.solgenomics.net

^3^https://swissmodel.expasy.org/

## References

[ref1] Ali R. Ma W. Lemtiri-Chlieh F. Tsaltas S. Berkowitz G. A. (2007). Death don’t have no mercy and neither does calcium: *Arabidopsis* CYCLIC NUCLEOTIDE GATED CHANNEL2 and innate immunity. Plant Cell 19, 1081–1095. 10.1105/tpc.106.045096, PMID: 17384171 PMC1867353

[ref2] Arnao M. B. Hernández-Ruiz J. (2006). The physiological function of melatonin in plants. Plant Signal. Behav. 1, 89–95. 10.4161/psb.1.3.2640, PMID: 19521488 PMC2635004

[ref001] Arnao M. B. Hernández-Ruiz J. (2015). Functions of melatonin in plants: a review. J. Pineal Res. 59, 133–150. 10.1111/jpi.12253 26094813

[ref3] Asai S. Yoshioka H. (2009). Nitric oxide as a partner of reactive oxygen species participates in disease resistance to necrotrophic pathogen *Botryis cinerea* in *Nicotiana benthamiana*. Mol. Plant-Microbe Interact. 22, 619–629. 10.1094/MPMI-22-6-0619, PMID: 19445587

[ref4] Bonilla E. Valero N. Chacín-Bonilla L. Medina-Leendertz S. J. (2004). Melatonin and viral infections. J. Pineal Res. 36, 73–79. 10.1046/j.1600-079X.2003.00105.x, PMID: 14962057 PMC7166828

[ref5] Brainard G. C. Hanifin J. P. Greeson G. Gerner E. Rollag M. D. (2011). Action spectrum for melatonin regulation in humans: evidence for a novel circadian photoreceptor. J. Neurosci. 21, 6405–6412. 10.1523/JNEUROSCI.21-16-06405.2001 PMC6763155 11487664

[ref6] Bright J. Desikan R. Hancock J. T. Weir I. S. Neill S. J. (2006). ABA induced NO generation and stomatal closure in Arabidopsis are dependent on H_2_O_2_ synthesis. Plant J. 45, 113–122. 10.1111/j.1365-313X.2005.02615.x, PMID: 16367958

[ref7] Byeon Y. Back K. (2014). An increase in melatonin in transgenic rice causes pleiotropic phenotypes, including enhanced seedling growth, delayed flowering, and low grain yield. J. Pineal Res. 56, 408–414. 10.1111/jpi.12129, PMID: 24571270

[ref9] Byeon Y. Lee H. Y. Lee K. Park S. Back K. (2014a). Cellular localization and kinetics of the rice melatonin biosynthetic enzymes SNAT and ASMT. J. Pineal Res. 56, 107–114. 10.1111/jpi.12103 24134674

[ref8] Byeon Y. Park S. Lee H. Kim Y. Back K. (2014b). Elevated production of melatonin in transgenic rice seeds expressing rice tryptophan decarboxylase. J. Pineal Res. 56, 275–282. 10.1111/jpi.12120 24433490

[ref10] Carrillo-Vico A. Lardone P. J. Alvarez-Sanchez N. Rodríguez-Rodríguez A. Guerrero J. M. (2013). Melatonin: buffering the immune system. Int. J. Mol. 14, 8638–8683. 10.3390/ijms14048638, PMID: 23609496 PMC3645767

[ref11] Chen X. Mou Y. Ling J. Wang N. Wang X. Hu J. (2015). Cyclic dipeptides produced by fungus *Eupenicillium brefeldianum* HMP-F96 induced extracellular alkalinization and H_2_O_2_ production in tobacco cell suspensions. World J. Microbiol. Biotechnol. 31, 247–253. 10.1007/s11274-014-1759-0, PMID: 25344087

[ref12] Chen Y. L. Huang R. F. Xiao Y. M. Lu P. Chen J. Wang X. C. (2004). Extracellular calmodulin-induced stomatal closure is mediated by heterotrimeric G protein and H_2_O_2_. Plant Physiol. 136, 4096–4103. 10.1104/pp.104.047837, PMID: 15557100 PMC535840

[ref13] Cheng-Hong C. Xin-Wang T. Heng-Chang Z. (2009). Progress on synthesis of melatonin. Food Drug. 11, 61–64.

[ref14] Ding F. Wang G. Wang M. Zhang S. (2018). Exogenous melatonin improves tolerance to water deficit by promoting cuticle formation in tomato plants. Molecules 23:1605. 10.3390/molecules23071605, PMID: 30004432 PMC6099739

[ref15] Dollins A. B. Zhdanova I. V. Wurtman R. J. Lynch H. J. Deng M. H. (1994). Effect of inducing nocturnal serum melatonin concentrations in daytime on sleep, mood, body temperature, and performance. Proc. Natl. Acad. 91, 1824–1828. 10.1073/pnas.91.5.1824, PMID: 8127888 PMC43256

[ref16] Dubbels R. Reiter R. J. Klenke E. Goebel A. Schnakenberg E. Ehlers C.. (1995). Melatonin in edible plants identified by radioimmunoassay and by high performance liquid chromatography-mass spectrometry. J. Pineal Res. 18, 28–31. 10.1111/j.1600-079X.1995.tb00136.x, PMID: 7776176

[ref17] Falcón J. Besseau L. Fuentès M. Sauzet S. Magnanou E. Boeuf G.. (2009). Structural and functional evolution of the pineal melatonin system in vertebrates. Ann. N. Y. Acad. 1163, 101–111. 10.1111/j.1749-6632.2009.04435.x 19456332

[ref18] Feng B. Shan L. (2014). ROS open roads to roundworm infection. Sci. Signal. 7:pe10. 10.1126/scisignal.2005273, PMID: 24714569 PMC4391739

[ref19] Garcia-Brugger A. Lamotte O. Vandelle E. Bourque S. Lecourieux D. Poinssot B.. (2006). Early signaling events induced by elicitors of plant defenses. Mol. Plant-Microbe Interact. 19, 711–724. 10.1094/MPMI-19-0711, PMID: 16838784

[ref20] Hattori A. Migitaka H. Iigo M. Itoh M. Yamamoto K. Ohtani-Kaneko R.. (1995). Identification of melatonin in plants and its effects on plasma melatonin levels and binding to melatonin receptors in vertebrates. Biochem. Mol. Biol. Int. 35, 627–634. PMID: 7773197

[ref21] Jian C. Mohan R. Zhang Y. Li M. Chen H. Palmer I. A.. (2019). NPR1 promotes its own and target gene expression in plant defense by recruiting CDK8. Plant Physiol. 181, 289–304. 10.1104/pp.19.00124 31110139 PMC6716257

[ref22] Jian C. Zhang J. Kong M. Freeman A. Chen H. Liu F.. (2021). More stories to tell: NONEXPRESSOR OF PATHOGENESIS-RELATED GENES1, a salicylic acid receptor. Plant Cell Environ. 44, 1716–1727. 10.1111/pce.14003 33495996

[ref23] Jian W. Dong-Xu L. Jia-Rong Z. Chi S. (2018). Phytomelatonin receptor PMTR1-mediated signaling regulates stomatal closure in *Arabidopsis thaliana*. J. Pineal Res. 65:e12500. 10.1111/jpi.12500 29702752

[ref24] La Camera S. Gouzerh G. Dhondt S. Hoffmann L. (2004). Metabolic reprogramming in plant innate immunity the contributions of phenylpropanoid and oxylipin pathways. Immunol. Rev. 198, 267–284. 10.1111/j.0105-2896.2004.0129.x, PMID: 15199968

[ref25] Lee H. Byeon Y. Back K. (2014). Melatonin as a signal molecule triggering defense responses against pathogen attack in *Arabidopsis* and tobacco. J. Pineal Res. 57, 262–268. 10.1111/jpi.12165, PMID: 25099383

[ref26] Lee H. Byeon Y. Tan D. X. Reiter R. J. Back K. (2015). Arabidopsis serotonin N-acetyltransferase knockout mutant plants exhibit decreased melatonin and salicylic acid levels resulting in susceptibility to an avirulent pathogen. J. Pineal Res. 58, 291–299. 10.1111/jpi.12214, PMID: 25652756

[ref27] Lee S. Choi H. Suh S. Doo I. S. Oh K. Y. Choi E. J. (1999). Oligogalacturonic acid and chitosan reduce stomatal aperture by inducing the evolution of reactive oxygen species from guard cells of tomato and *Commelina communis*. Plant Physiol. 121, 147–152. 10.1104/pp.121.1.147, PMID: 10482669 PMC59362

[ref28] Lerner A. Case J. Takahashi Y. Lee T. H. (1958). Isolation of melatonin, the pineal gland factor that lightens melanocytes. J. Am. Chem. Soc. 80:2587. 10.1021/ja01543a060

[ref29] Levoye A. Dam J. Ayoub M. A. Guillaume J.-L. Couturier C. Delagrange P.. (2006). The orphan GPR50 receptor specifically inhibits MT1 melatonin receptor function through heterodimerization. EMBO J. 25, 3012–3023. 10.1038/sj.emboj.7601193, PMID: 16778767 PMC1500982

[ref30] Li M. Q. Hasan M. K. Li C. X. Ahammed G. J. Xia X.-J. Shi K.. (2016b). Melatonin mediates seleniuminduced tolerance to cadmium stress in tomato plants. J. Pineal Res. 61, 291–302. 10.1111/jpi.12346, PMID: 27264631

[ref31] Liang C. Zheng G. Li W. Wang Y. Hu B. Wang H.. (2015). Melatonin delays leaf senescence and enhances salt stress tolerance in rice. J. Pineal Res. 59, 91–101. 10.1111/jpi.12243, PMID: 25912474

[ref32] Liu Y. Schiff M. Marathe R. Dinesh-Kumar S. P. (2002). Tobacco Rar1. EDS1 and NPR1/NIM1 like genes are required for N-mediated resistance to tobacco mosaic virus. Plant J. 30, 415–429. 10.1046/j.1365-313X.2002.01297.x, PMID: 12028572

[ref33] Mandal M. K. Suren H. Ward B. Boroujerdi A. Kousik C. (2018). Differential roles of melatonin in plant-host resistance and pathogen suppression in cucurbits. J. Pineal Res. 65:e12505. 10.1111/jpi.12505, PMID: 29766569

[ref34] Murashige T. Skoog F. (1962). A revised medium for rapid growth and bioassays with tobacco tissue cultures. Physiol. Plant. 15, 473–497. 10.1111/j.1399-3054.1962.tb08052.x

[ref35] Murch S. Saxena P. (2002). Melatonin: a potential regulator of plant growth and development? In vitro. Cell. Dev. Biol. Plant. 38, 531–536. 10.1079/IVP2002333

[ref36] Nabavi S. M. Nabavi S. F. Sureda A. Xiao J. Dehpour A. R. Shirooie S.. (2019). Anti-inflammatory effects of melatonin: a mechanistic review. Crit. Rev. Food Sci. Nutr. 59, 4–16. 10.1080/10408398.2018.1487927 29902071

[ref37] Nosjean O. Ferro M. Coge F. Beauverger P. Henlin J. M. Lefoulon F.. (2000). Identification of the melatonin-binding site MT3 as the quinone reductase 2. J. Biol. Chem. 275, 31311–31317. 10.1074/jbc.M005141200, PMID: 10913150

[ref38] Oh S. K. Kwon S. Y. Choi D. (2014). Rpi-blb2-mediated hypersensitive cell death caused by *Phytophthora infestans* AVRblb2 requires SGT1, but not EDS1, NDR1, salicylic acid-, jasmonic acid-, or ethylene-mediated signaling. Plant Pathol. J. 30, 254–260. 10.5423/PPJ.OA.03.2014.0027, PMID: 25289011 PMC4181110

[ref39] Park S. Lee K. Kim Y.-S. Back K.. (2012). Tryptamine5-hydroxylase-deficient *Sekiguchi* rice induces synthesis of 5-hydroxytryptophan and N-acetyltryptamine but decreases melatonin biosynthesis during senescence process of detached leaves. J. Pineal Res. 52, 211–216. 10.1111/j.1600-079X.2011.00930.x, PMID: 21884550

[ref40] Pasqualini S. Paolocci F. Borgogni A. Morettini R. Ederli L. (2007). The overexpression of an alternative oxidase gene triggers ozone sensitivity in tobacco plants. Plant Cell Environ. 30, 1545–1556. 10.1111/j.1365-3040.2007.01730.x, PMID: 17944819

[ref41] Peart J. R. Lu R. Sadanandom A. Malcuit I. Moffett P. Brice D. C.. (2002). Ubiquitin ligase-associated protein SGT1 is required for host and nonhost disease resistance in plants. Proc. Natl. Acad. 99, 10865–10869. 10.1073/pnas.152330599, PMID: 12119413 PMC125064

[ref42] Pieterse C. M. Leon-Reyes A. Van der Ent S. Van Wees S. C. (2009). Networking by small-molecule hormones in plant immunity. Nat. Chem. Biol. 5, 308–316. 10.1038/nchembio.164, PMID: 19377457

[ref43] Regodon S. Martín-Palomino P. Fernández-Montesinos R. Herrera J. L. Carrascosa-Salmoral M. P. Píriz S.. (2005). The use of melatonin as a vaccine agent. Vaccine 23, 5321–5327. 10.1016/j.vaccine.2005.07.003, PMID: 16055232

[ref44] Reiter R. (1999). Pineal melatonin: cell biology of its synthesis and of its physiological interactions. Endocr. Rev. 12, 151–180. 10.1210/edrv-12-2-151 1649044

[ref45] Reiter R. Tan D. Fuentesbroto L. (2010). Melatonin: a multitasking molecule. Prog. Brain Res. 181, 127–151. 10.1016/S0079-6123(08)81008-4 20478436

[ref47] Reppert S. M. Godson C. Mahle C. D. Weaver D. R. Slaugenhaupt S. A. Gusella J. F.. (1995). Molecular characterization of a second melatonin receptor expressed in human retina and brain: the Mel1b melatonin receptor. Proc. Natl. Acad. 92, 8734–8738. 10.1073/pnas.92.19.8734 PMC41041 7568007

[ref46] Reppert S. M. Weaver D. R. Ebisawa T. (1994). Cloning and characterization of a mammalian melatonin receptor that mediates reproductive and circadian responses. Neuron 13, 1177–1185. 10.1016/0896-6273(94)90055-8, PMID: 7946354

[ref48] Rodriguez C. Mayo J. C. Sainz R. M. Antolin I. Herrera F. Martín V.. (2004). Regulation of antioxidant enzymes: a significant role for melatonin. J. Pineal Res. 36, 1–9. 10.1046/j.1600-079X.2003.00092.x, PMID: 14675124

[ref49] Samuel M. A. Hall H. Krzymowska M. Drzewiecka K. Hennig J. Ellis B. E. (2005). SIPK signaling controls multiple components of harpin-induced cell death in tobacco. Plant J. 42, 406–416. 10.1111/j.1365-313X.2005.02382.x, PMID: 15842625

[ref50] Sathiyabama M. Balasubramanian R. (1998). Chitosan induces resistance components in *Arachis hypogaea* against leaf rust caused by *Puccinia arachidis* Speg. Crop Prot. 17, 307–313. 10.1016/S0261-2194(98)00017-9

[ref51] Schuhegger R. Ihring A. Gantner S. Bahnweg G. Knappe C. Vogg G.. (2006). Induction of systemic resistance in tomato by N-acyl-Lhomoserine lactone-producing rhizosphere bacteria. Plant Cell Environ. 29, 909–918. 10.1111/j.1365-3040.2005.01471.x, PMID: 17087474

[ref52] Shi H. Jiang C. Ye T. Tan D. X. Reiter R. J. Zhang H.. (2015b). Comparative physiological, metabolomic, and transcriptomic analyses reveal mechanisms of improved abiotic stress resistance in bermudagrass [*Cynodon dactylon* (L). Pers.] by exogenous melatonin. J. Exp. Bot. 66, 681–694. 10.1093/jxb/eru373, PMID: 25225478 PMC4321537

[ref53] Shi H. Qian Y. Tan D. X. Reiter R. J. He C. (2015c). Melatonin induces the transcripts of CBFDREB1s and their involvement in both abiotic and biotic stresses in *Arabidopsis*. J. Pineal Res. 59, 334–342. 10.1111/jpi.12262, PMID: 26182834

[ref54] Shi H. Tan D. X. Reiter R. J. Ye T. Yang F. Chan Z. (2015d). Melatonin induces class A1 heat-shock factors (HSFA1s) and their possible involvement of thermotolerance in *Arabidopsis*. J. Pineal Res. 58, 335–342. 10.1111/jpi.12219, PMID: 25711624

[ref55] Sun Q. Zhang N. Wang J. Zhang H. Li D. Shi J.. (2015). Melatonin promotes ripening and improves quality of tomato fruit during postharvest life. J. Exp. Bot. 66, 657–668. 10.1093/jxb/eru332, PMID: 25147270 PMC4321535

[ref56] Tan D. X. Manchester L. C. Liu X. Rosales-Corral S. A. Acuna-Castroviejo D. Reiter R. J.. (2013). Mitochondria and chloroplasts as the original sites of melatonin synthesis: a hypothesis related to melatonin’s primary function and evolution in eukaryotes. J. Pineal Res. 54, 127–138. 10.1111/jpi.12026, PMID: 23137057

[ref57] Tassel D. V. Roberts N. O’Neill S. (1995). Melatonin from higher plants: isolation and identification of N-acetyl-5-methoxytryptamine. Plant Physiol. 108:101.

[ref58] Vielma J. R. Bonilla E. Chacín-Bonilla L. Mora M. Medina-Leendertz S. Bravo Y.. (2014). Effects of melatonin on oxidative stress, and resistance to bacterial, parasitic, and viral infections: a review. Acta Trop. 137, 31–38. 10.1016/j.actatropica.2014.04.021, PMID: 24811367

[ref59] Wang P. Yin L. Liang D. Li C. Ma F. Yue Z.. (2012). Delayed senescence of apple leaves by exogenous melatonin treatment: toward regulating the ascorbate-glutathione cycle. J. Pineal Res. 53, 11–20. 10.1111/j.1600-079X.2011.00966.x, PMID: 21988707

[ref60] Wei W. Li Q. T. Chu Y. N. Reiter R. J. Yu X.-M. Zhu D.-H.. (2015). Melatonin enhances plant growth and abiotic stress tolerance in soybean plants. J. Exp. Bot. 66, 695–707. 10.1093/jxb/eru392, PMID: 25297548 PMC4321538

[ref61] Yin L. Wang P. Li M. Ke X. Li C. Liang D.. (2013). Exogenous melatonin improves Malus resistance to Marssonina apple blotch. J. Pineal Res. 54, 426–434. 10.1111/jpi.12038, PMID: 23356947

[ref002] Yoshioka H. Asai S. Yoshioka M. Kobayashi M. (2009). Molecular mechanisms of generation for nitric oxide and reactive oxygen species, and role of the radical burst in plant immunity. Mol. Cells. 28:321. 10.1007/s10059-009-0156-2 19830396

[ref62] Zhang H. Dong S. Wang M. Wang W. Song W. Dou X.. (2010). The role of vacuolar processing enzyme (VPE) from *Nicotiana benthamiana* in the elicitor-triggered hypersensitive response and stomatal closure. J. Exp. Bot. 61, 3799–3812. 10.1093/jxb/erq189, PMID: 20603283 PMC2921209

[ref63] Zhang H. Fang Q. Zhang Z. Wang Y. Zheng X. (2009). The role of respiratory burst oxidase homologues in elicitor-induced stomatal closure and hypersensitive response in *Nicotiana benthamiana*. J. Exp. Bot. 60, 3109–3122. 10.1093/jxb/erp146, PMID: 19454596 PMC2718215

[ref64] Zhang N. Sun Q. Zhang H. Zhao C. Li L. Chen M. (2015). Roles of melatonin in abiotic stress resistance in plants. J. Exp. Bot. 66, 647–656. 10.1093/jxb/eru336, PMID: 25124318

[ref65] Zhang N. Zhang H. Zhao B. Sun Q.-Q. Cao Y.-Y. Li R.. (2014). The RNA-seq approach to discriminate gene expression profiles in response to melatonin on cucumber lateral root formation. J. Pineal Res. 56, 39–50. 10.1111/jpi.12095, PMID: 24102657

[ref66] Zhu F. Xi D. H. Yuan S. Xu F. Zhang D. W. Lin H. H. (2014). Salicylic acid and jasmonic acid are essential for systemic resistance against tobacco mosaic virus in *Nicotiana benthamiana*. Mol. Plant-Microbe Interact. 27, 567–577. 10.1094/MPMI-11-13-0349-R, PMID: 24450774

[ref67] Zuppini A. Baldan B. Millioni R. Favaron F. Navazio L. Mariani P. (2004). *Chitosan* induces Ca21-mediated programmed cell death in soybean cells. New Phytol. 161, 557–568. 10.1046/j.1469-8137.2003.00969.x, PMID: 33873499

